# Neural and affective responses to prolonged eye contact with parents in depressed and nondepressed adolescents

**DOI:** 10.3758/s13415-024-01169-w

**Published:** 2024-02-22

**Authors:** Mirjam C. M. Wever, Geert-Jan Will, Lisanne A. E. M. van Houtum, Loes H. C. Janssen, Wilma G. M. Wentholt, Iris M. Spruit, Marieke S. Tollenaar, Bernet M. Elzinga

**Affiliations:** 1 Department of Clinical Psychology, Faculty of Social and Behavioral Sciences, Leiden University, 2300 RB Leiden, the Netherlands; 2 Leiden Institute for Brain and Cognition, Leiden, the Netherlands; 3 Department of Clinical Psychology, Utrecht University, Utrecht, the Netherlands

**Keywords:** Prolonged eye contact, Major depressive disorder, Parent–child bonding, Nonverbal social cues, fMRI, Eye tracking

## Abstract

Eye contact improves mood, facilitates connectedness, and is assumed to strengthen the parent–child bond. Adolescent depression is linked to difficulties in social interactions, the parent–child bond included. Our goal was to elucidate adolescents’ affective and neural responses to prolonged eye contact with one’s parent in nondepressed adolescents (HC) and how these responses are affected in depressed adolescents. While in the scanner, 59 nondepressed and 19 depressed adolescents were asked to make eye contact with their parent, an unfamiliar peer, an unfamiliar adult, and themselves by using videos of prolonged direct and averted gaze, as an approximation of eye contact. After each trial, adolescents reported on their mood and feelings of connectedness, and eye movements and BOLD-responses were assessed. In HCs, eye contact boosted mood and feelings of connectedness and increased activity in inferior frontal gyrus (IFG), temporal pole, and superior frontal gyrus. Unlike HCs, eye contact did not boost the mood of depressed adolescents. While HCs reported increased mood and feelings of connectedness to the sight of their parent versus others, depressed adolescents did not. Depressed adolescents exhibited blunted overall IFG activity. These findings show that adolescents are particularly sensitive to eye contact and respond strongly to the sight of their parents. This sensitivity seems to be blunted in depressed adolescents. For clinical purposes, it is important to gain a better understanding of how the responsivity to eye contact in general and with their parents in particular, can be restored in adolescents with depression.

**Supplementary information:**

The online version contains supplementary material available at 10.3758/s13415-024-01169-w.

## Introduction

Eye contact facilitates strong feelings of connectedness with others (Emery, 2000; Hietanen, 2018) and particularly *prolonged* eye contact has been associated with stronger positive affect (Kuzmanovic et al., 2009; Wever et al., 2022). Within the parent–child context, eye contact constitutes one of the first acts of reciprocity between a parent and child after birth and is considered an important facilitator for a strong parent–child bond (Robson, 1967). Being able to draw the attention of one’s parent by making eye contact enables infants to signal their physical and emotional needs to their parent. At the same time, the rewarding nature of positive affect through eye contact with a child is thought to reinforce sensitive caregiving behavior in parents (Robson, 1967). In a previous study, we showed that eye contact between a parent and child is still relevant during adolescence, because parents reported a better mood and feel more connected in response to eye contact with their adolescent child compared with unfamiliar others (Wever et al., 2022). However, how *adolescents* respond to eye contact with their parent at the neural and affective level has remained unclear.

Adolescence is characterized by substantial changes in the socioemotional domain (Crone & Dahl, 2012). Adolescents show enhanced sensitivity to social events, with negative social experiences (e.g., rejection) particularly inducing negative feelings (Vijayakumar et al., 2017; Will et al., 2016). Because eye contact is one of the most common nonverbal social signals to initiate social contact and maintain connections with others, it is plausible that adolescents are particularly sensitive to eye contact. Despite the increased importance of the peers during adolescence, parental support remains highly associated with adolescents’ wellbeing and mental health (Baumrind, 1991; Yap et al., 2014). While there is some work on neural processing of eye contact in adults, no previous studies have examined neural and affective responses to eye contact during adolescence. Moreover, it is unclear whether these responses are moderated by individual differences in the parent–child relationship.

Enhanced sensitivity to social cues during adolescence has been proposed to heighten the risk for developing depression, especially when adolescents are exposed to chronic social adversity, such as social exclusion or bullying victimization (Crone & Dahl, 2012; Dahl, 2004; Giedd & Rapoport, 2010; Hankin & Abramson, 2001; Wilson et al., 2015). Common characteristics of adolescent depression include difficulties in the social domain, such as social isolation and dysfunctional interpersonal relationships (Hammen, 2009; Hammen et al., 2008). These difficulties may lead to lower levels of relationship satisfaction with both parents and peers (Babore et al., 2016; Branje et al., 2010; Heaven et al., 2004; Sheeber et al., 2001). One hypothesis is that depressed adolescents who suffer from difficulties in interpersonal contact with others may exhibit altered neural responses in networks supporting social cognition. In addition, previous studies in adults have shown, for example, that depressed adults interpret neutral faces as more negative (Rubinow & Post, 1992; Surguladze et al., 2004), engage less in eye contact, use fewer gestures (Hames et al., 2013; Kazdin et al., 1985; Suffel et al., 2020), and show less modulation in their voice compared with nondepressed adults (Youngren & Lewinsohn, 1980). Moreover, adolescents with depression perceive their parents as less warm and more critical, and report a lower parent–child relationship quality compared with nondepressed adolescents (Branje et al., 2010; Sheeber & Sorensen, 1998; Yap et al., 2010). 

This study therefore examined adolescents’ general responses to prolonged eye contact with their parent in adolescents without psychopathology (goal 1) to acquire a normative benchmark. In addition, we examined whether these responses differ between depressed and nondepressed adolescents (goal 2). This might elucidate processes regarding difficulties in interpersonal functioning in depressed adolescents and whether this concerns interactions in general or is specifically relevant to the parent-child context. To examine whether adolescents’ responses are unique to the parent–child relationship, we compared responses to eye contact with their parents with adolescents’ responses to eye contact with an unfamiliar adult. In order to see whether adolescents show altered responses to their parents compared with an unknown peer, we examined how they respond to making eye contact with an unfamiliar peer of the same age and sex. We used a novel eye contact task (validated in adults; Wever et al., 2022) in which adolescents are presented with personalized videos of prolonged direct and averted gaze, as an approximation of eye contact. Nondepressed adolescents were, similar to adults (Wever et al., 2022), hypothesized to have a better mood and feel more connected after making prolonged eye contact with others. Moreover, we expect adolescents to exhibit differential neural responses when looking at their parent versus unfamiliar peers or adults in brain regions associated with mentalizing (i.e., dmPFC, TPJ) and familiarity and attachment (i.e., IFG, fusiform gyrus) (Feldman, 2017; Laurita et al., 2019). This is not only based on previous neuroimaging findings on eye contact between parents and adolescents (Wever et al., 2022) but also on a broader literature on differential salience between familiar and nonfamiliar others (Natu & O’Toole, 2011; Ramon & Gobbini, 2018). With regard to the depressed adolescents we expect that these adolescents feel less connected and report a lower mood in response to eye contact compared with nondepressed adolescents, independent of target identity. We do expect that, similar to nondepressed adolescents, depressed adolescents feel more connected and report a better mood when seeing their parent versus an unfamiliar other (peer or adult), but that this effect is less pronounced compared with nondepressed adolescents. Lastly, we explored whether depressed and nondepressed adolescents differ in their neural and gaze responses to eye contact with their parents and unfamiliar others. All study measures, hypotheses, and analyses were preregistered before data analyses (https://osf.io/p6r28/, where the current study relates to Part 1 of the preregistration, and Part 2 will be reported elsewhere).

## Method

### Participants

Adolescents and their parents participated in the context of the RE-PAIR study: Relations and Emotions in Parent-Adolescent Interaction Research. This study compared families with an adolescent with major depressive disorder (MDD) or dysthymia to families with an adolescent without psychopathology. Families included in the study were Dutch speaking, adolescents were aged between 11 and 17 years at study inclusion, and lived with at least one of their parents/caregivers. Families with an adolescent without psychopathology (healthy controls; HC) were included if they were not diagnosed with a (neuro)psychiatric disorder in the 2 years leading up to the study and had no lifetime diagnosis of MDD/dysthymia. Families with an adolescent with MDD or dysthymia (DEP) were included if the adolescent met full criteria on one of these primary diagnoses, verified with the Kiddie-Schedule for Affective Disorders and Schizophrenia Present and Lifetime version (K-SADS; Kaufman et al. (1996)). They could not participate if the adolescent met criteria for a comorbid psychosis, substance use disorder, or mental retardation. Exclusion criteria for the functional magnetic resonance imaging (fMRI) part of the study were incompatibilities with MRI scanning. Because a depressed mood is one of the primary characteristics in both MDD and dysthymia according to the DSM-IV, both adolescents with a diagnosis of MDD or dysthymia were included in our “depressed group”. This grouping of adolescents with both MDD and dysthymia diagnoses was supported by a t-test showing no significant difference in depression severity assessed with the PHQ-9 (*t*(17) =  − 0.60, *p* = 0.555).

 Sixty-three HC and 22 DEP adolescents participated in the fMRI part of the study. Four HCs were excluded: three because of scanner artefacts, and one because of an (a posteriori discovered) depression score in the clinical range according to the Patient Health Questionnaire (i.e., PHQ-score of 18), which was preregistered as an exclusion criterion. Three DEP adolescents were excluded: one because of scanner artefacts, one because of claustrophobia, and one because of distress related to exposure to participant’s own videos. This resulted in a final sample of 59 HC and 19 DEP adolescents (MDD:* n* = 15, dysthymia: *n* = 4) performing the eye contact task in the MRI scanner (see Table 1 for demographics and clinical characteristics). This is a subsample of the full study sample of the RE-PAIR study, but there were no significant differences between the samples in terms of group differences on age, sex, depression severity, and puberty score. It is worth noting that the age of the participants in our study only covers a part of the typical age range of adolescence. The age range in our study (11 − 17 years) was related to our inclusion criteria that all adolescents needed to attend at least secondary school, which in the Netherlands starts at the age of 11 or 12 years, and still had to live with (one of) their parents. Furthermore, the mean age of the adolescents in this study was 16, showing that the age distribution was relatively skewed. However, this skewed distribution reflects the age around which the depressed adolescents seek treatment at the mental health care facilities that we cooperated with for recruitment of depressed adolescents and their parents. We matched participants in our control group to the depressed adolescents in terms of age and sex.

**Table 1:** Demographics and clinical characteristics of depressed (DEP) and nondepressed (HC) adolescents

	HC	DEP	HC vs. DEP^1^	
Mean (SD) /*n* (%)	(*n* = 59)	(*n* = 19)	*t* / χ^2^	*p*
Age adolescents, years	16.20 (1.21)	16.04 (1.50)	0.45	0.650
Age parents, years	49.90 (5.17)	50.82 (4.81)	− 0.69	0.495
Sex adolescents				
*Girls, n (%)*	39 (66.1)	15 (79.0)	1.11	0.291
Current educational level			2.00	0.788
*Lower vocational*	7 (11.86)	3 (15.79)	-	-
*Higher vocational*	17 (28.81)	3 (15.79)	-	-
*Pre-university*	28 (47.46)	9 (47.37)	-	-
*Secondary vocational*	5 (8.48)	3 (15.79)	-	-
*Higher professional*	2 (3.39)	1 (5.26)	-	-
Handedness (EHI)				
*Right-handed*, *n* (%)	54 (91.53)	18 (94.74)	0.21	1.00
Pubertal development (PDS)	3.26 (0.63)	3.47 (0.62)	− 1.27	0.209
Depression severity (PHQ-9)	4.32 (2.54)	17.90 (4.05)	− 17.32	< 0.001
Anxiety severity (SCARED)	12.32 (7.27)	34.21 (9.21)	− 10.67	< 0.001
Parental-child bonding (PBI)				
*Care*	30.90 (5.12)	26.47 (5.99)	3.13	0.003
*Overprotection*	3.41 (2.24)	7.26 (4.07)	− 5.22	< 0.001
*Lack of autonomy support*	3.52 (2.57)	5.05 (4.55)	− 1.84	0.070

*Note.* DEP = depressed adolescents; EHI = Edinburgh Handedness Inventory (Oldfield, 1971); HC = healthy control adolescents; PBI = Parental Bonding Instrument (Parker et al., 1979); PDS = Pubertal Development Scale (Petersen et al., 1988); PHQ-9 = Patient Health Questionnaire (Kroenke et al., 2001); SCARED = Screen for Child Anxiety Related Emotional Disorders (Simon & Bögels, 2009); SD = standard deviation. ^1^*p*-values were obtained using independent samples *t*-tests or chi-square comparisons between depressed and nondepressed adolescents

The study was approved by the medical ethical committee of the Leiden University Medical Centre (LUMC; P17.241) and was performed in accordance with the declaration of Helsinki and the Dutch Medical Research Involving Human Subjects Act (WMO).

### Procedure

Families with an HC adolescent were recruited via public advertisements and (online) social media, including Facebook and advertisement in the monthly magazine of the Royal Dutch Touring Club (ANWB). Families with a depressed adolescent were recruited via mental health facilities. Parents and adolescents were briefed about the study and underwent a comprehensive telephone screening during which family circumstances and informed consent were discussed and adolescents were prescreened for (a history of) psychiatric disorders. Families were invited for two appointments: An assessment day in the lab and an MRI session on a separate day. Before the first appointment, adolescents were asked to fill out an online questionnaire battery, including demographics and clinical and cognitive measures (see Supplement 4 for details). During the first appointment, families performed parent–adolescent interaction tasks and completed additional questionnaires. During the second appointment, adolescents underwent an MRI scan at the LUMC in Leiden, the Netherlands. Before and after the scan, adolescents completed a set of questionnaires, received instructions about the MRI tasks, and performed some practice trials. Adolescents performed four tasks in the MRI scanner: The eye contact task as described below, the parental social feedback task (van Houtum et al., 2022), a peer evaluation task, and an autobiographical memory task. Upon completion of the MRI scans, adolescents were fully debriefed about the goals of the study and received a monetary compensation and travel allowance. All participants provided written, informed consent for each individual testing day. The median of days between the first and second appointment was 42 days (range 7–265 days) and did not significantly differ between depressed and nondepressed adolescents (*t*(76) =  − 0.77, *p* = 0.444).

### Measures and materials

#### Eye contact task

**Fig. 1:**
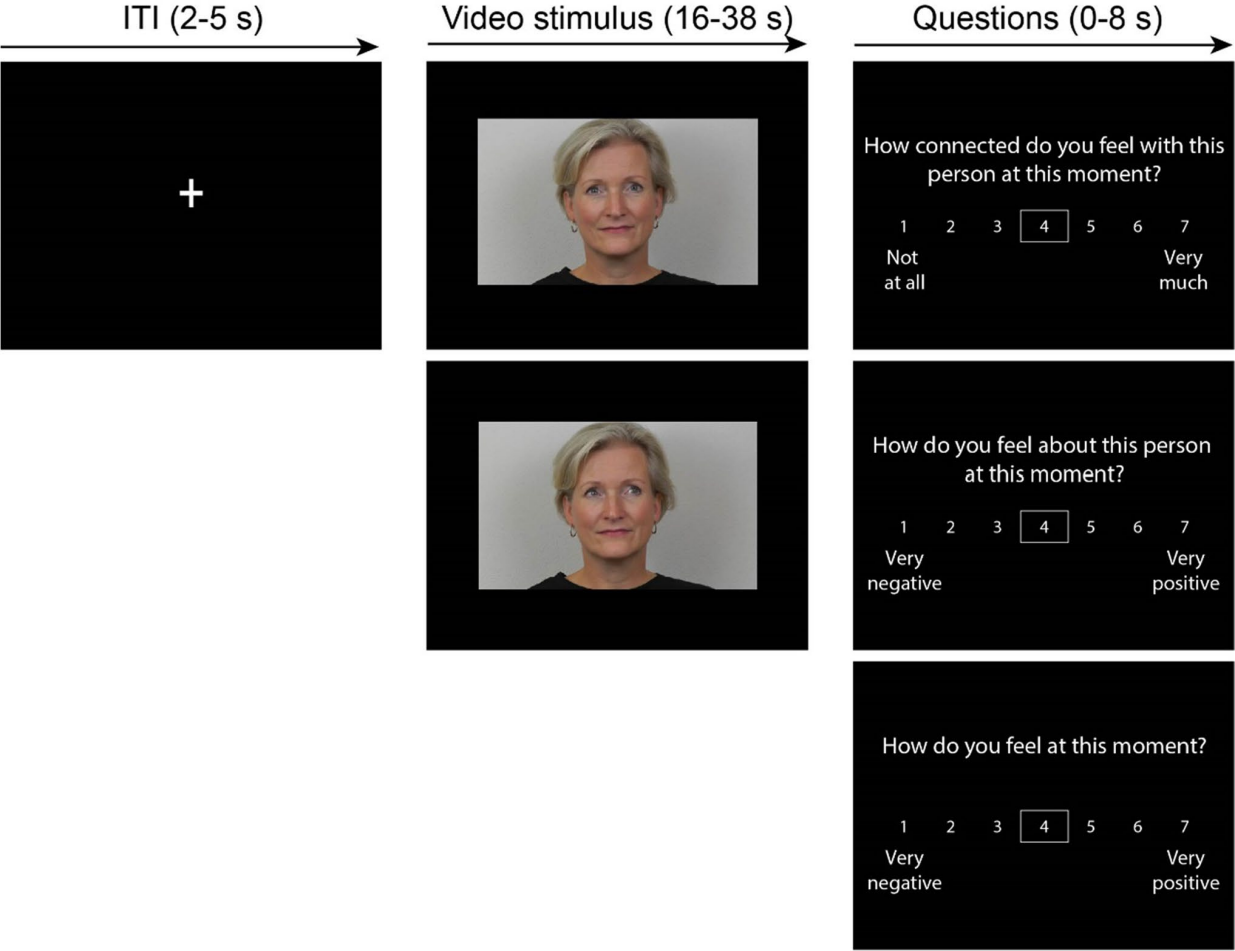
Displays and timings of video stimuli of an unfamiliar adult with a direct and averted gaze in the eye contact task. Videos of adolescents’ parent were contrasted with videos of an unfamiliar same-sex parent, an unfamiliar peer (same-sex adolescent), and videos of the self

To assess neural and affective responses to prolonged eye contact, adolescents performed the “eye contact task” (see Wever et al. (2022) and Fig. 1 for an overview of the task). During this task, adolescents were shown prerecorded video stimuli of their parent, an unfamiliar peer, an unfamiliar adult, and themselves—all facing the camera. Each video contained a single target who looked straight into the camera (direct gaze) or averted their gaze to the left side of the camera (averted gaze), resulting in eight distinct conditions: Gaze direction (2 levels: Direct gaze, averted gaze) × target (4 levels: Parent, unfamiliar peer, unfamiliar adult, self). See Supplement 1 for details about preparation and presentation of the videos. We simultaneously recorded eye movements using an eye-tracker.

While in the scanner, adolescents were instructed to make eye contact with the targets in the videos. Each trial started with a fixation cross (2–5 s), after which they were presented with a video of one of the targets in one of the gaze directions for 16–38 s. Sex of the unfamiliar peer was matched to adolescents’ own sex and sex of the unfamiliar adult was matched to sex of the parent condition in the task. After watching each video, adolescents were asked to answer three questions; (1) “How connected do you feel with this person at this moment?”; (2) “How do you feel about this person at this moment?”; and (3) “How do you feel at this moment?” After the videos of themselves, adolescents only reported on their mood (question 3). Adolescents answered the questions on a Likert scale ranging from 1 (*not at all/very negative*) to 7 (*very much/very positive*) and were instructed to answer and confirm the question within 8 s. The questions were self-paced and participants could press any button to display a box around the middle option, and then press the button corresponding to their right index (to go left) and right middle finger (to go right) to move the box to their preferred answer. They could confirm their answer by pressing the button corresponding to their left index finger. Before the task, adolescents answered all abovementioned questions in response to pictures of each target with a direct gaze. We included this baseline measure to ascertain whether increases in affect could be attributed to making prolonged eye contact relative to baseline. Stimulus presentation and simultaneous eye movement recordings were conducted using E-Prime 2.0 software (PsychologySoftware Tools, Pittsburgh, PA) and the screen resolution was 1024 × 768 pixels. The videos were presented on the screen in 960 × 540 pixels.

### Data preprocessing and analyses

#### Affective and gaze responses

Adolescents’ affective and gaze responses were analyzed in *R* (R Core Team (2013), version 3.6.1), with the following packages: Lme4 for mixed model analyses, psych for descriptive statistics, and ggplot2 for data visualization (Bates et al., 2012; Revelle, 2012; Wickham et al., 2016). Questions that were not answered and/or confirmed were excluded from all affective response analyses, which resulted in 0.5% missing responses (11/2360 trials) in HC adolescents and 0.3% (2/760 trials) in DEP adolescents.

#### Eye-tracking analyses

Eye movements were recorded with a tower mounted monocular EyeLink 1000 Hz MRI-compatible eye tracker (SR Research Ltd., Mississauga, Ontario, Canada), placed inside the scanner bore. We used a customized MATLAB (MathWorks, Inc., Natick, MA, version 9.5) script to preprocess raw eye movement data into information on gaze position and duration. Using an established algorithm for face and facial feature detection (Viola & Jones, 2001), we created rectangular areas of interest (AOIs) around the left and right eye of the targets in all videos that were combined into a single AOI of the eye region for further analyses. The primary gaze measure was the percentage of dwell time within the eye region per video relative to the total video duration, in which dwell time is defined as the time spent looking within an AOI. The eye tracker was calibrated and validated using a nine-point calibration grid from EyeLink’s calibration protocol. Collection of gaze data of 25 adolescents (HC: *n* = 17, DEP: *n* = 8) was unsuccessful due to technical problems or a failed calibration procedure (e.g., because of sight deficiencies, participants wearing glasses, or participants having light-colored eyes). In addition, 19 trials of nine adolescents (HC: *n* = 6, DEP: *n* = 3) were excluded due to > 30% missing gaze data. This resulted in a final set of gaze data of 53 (of 78) adolescents (HC: *n* = 42; DEP: *n* = 11), including 829 trials (of 848; 2.2% missing).

#### fMRI data acquisition and neuroimaging analyses

MR images were acquired using a Philips 3.0 T Achieva MRI scanner equipped with a SENSE-32 channel head coil. For the eye contact task, T2*-weighted echo planar imaging was used and a structural 3D T1 scan was acquired (see Supplement 2 for details on scan parameters). MRI data were preprocessed and analyzed using SPM12 (Wellcome Trust Centre for Neuroimaging, University College London). Functional MR images were slice-time corrected, corrected for field-strength inhomogeneity’s using b0 field maps, unwarped and realigned, co-registered to subject-specific structural images, normalized to MNI space using the DARTEL toolbox (Ashburner, 2007), and smoothed using an 8-mm full width at half maximum isotropic Gaussian kernel. Raw and preprocessed data were checked for quality, registration, and movement. Average head movement per adolescent did not exceed 1 voxel (i.e., 3 mm) (*M* = 0.075 mm, SD = 0.083 mm, range 0.038–0.252 mm). We corrected for serial autocorrelations using a first order autoregressive model (AR(1)). Low-frequency signals were removed using a high-pass filter (cutoff = 128 s), and we included nuisance covariates to remove effects of run.

First, to examine neural responses to gaze direction, target, and their interaction within the sample of HC adolescents, we constructed a generalized linear mixed regression model with eight regressors, indicating cue onset for each condition and one regressor for onsets of subjective ratings. Cue onset regressors were defined from the onset of the video and modeled for its duration (16–38 s). The subjective rating regressor was defined from the onset of each question and modeled for the duration the question was displayed on the screen, including 1000 ms during which a “Too late!” screen was shown if adolescents did not answer within the set time period of 8000 ms (self-paced; M = 2946 ms; SD = 1259 ms; range = 749–9002 ms). Six motion parameters (based on the realignment parameters) were included to correct for head motion. Eight first-level SPM T-contrasts were specified, one for each condition (i.e., direct and averted gaze of parent, unfamiliar peer, unfamiliar adult, self). These T-contrast images were entered in a 2 × 4 full factorial ANOVA design with two within-subject factors (gaze direction and target). SPM F-maps were computed to assess main effects of gaze direction and target, and their interaction, followed up by post-hoc analyses between all conditions. Second, to examine group differences between DEP and HC adolescents, we entered previously described t-contrast in a 2 × 2 × 4 full factorial ANOVA design with one between-subject factor (group) and two within-subject factors (gaze direction and target). SPM F-maps were computed to assess main effects of group, gaze direction, and target, and their interactions (i.e., group × gaze direction, group × target, group × gaze direction × target), followed up by post-hoc analyses between all conditions.

At the second-level, we first performed region of interest (ROI) analyses by using independently defined functional ROIs (8-mm spheres MNI space) surrounding peak voxels of brain region based on previously found regions using the eye contact task in a sample of adults (Wever et al., 2022) (see Supplement 3). We used the MarsBar toolbox (Brett et al., 2002) to extract activity from five ROIs, i.e., left temporoparietal junction (TPJ), dorsomedial prefrontal cortex (dmPFC), bilateral inferior frontal gyrus (IFG), and right fusiform gyrus (FG). To assess the effects of group, gaze direction, target, and their interactions, we performed linear mixed regression analyses and post-hoc tests in *R*. All ROI analyses were Bonferroni corrected for the number of tests (*p* < 0.05/5). Thereafter, to explore blood oxygenation level-dependent (BOLD) responses in brain regions outside the ROIs, we performed complementary whole-brain analyses that were corrected for multiple comparisons with family-wise error cluster correction at *p* < 0.05 (with a cluster-forming threshold of *p* < 0.001).

To check whether results were not driven by differences in age, sex, handedness (only in fMRI analyses), and pubertal status of adolescents, we performed additional analyses to control for these variables (see Supplement 4 for details on associated questionnaires). A total number of eleven hypotheses were tested: Three for each affective response question; one for participants’ gaze responses (Supplement 7); five ROI analyses; and two whole-brain analyses. Our hypotheses were preregistered and were divided over three measurement levels (i.e., affective, gaze, and neural responses). Therefore, no corrections for multiple comparisons were made, except for the ROI analyses, which were Bonferroni corrected (as preregistered).

## Results

### Adolescents’ responses to prolonged eye contact in HC adolescents (Goal 1)

#### Affective responses

To examine adolescents’ affective responses to eye contact in HC adolescents and how this may vary as a function of target, we performed a generalized linear mixed regression model with gaze direction, target, and their interaction on adolescents’ affect ratings (i.e., mood, connectedness, feelings about the targets).

##### Mood

**Fig. 2:**
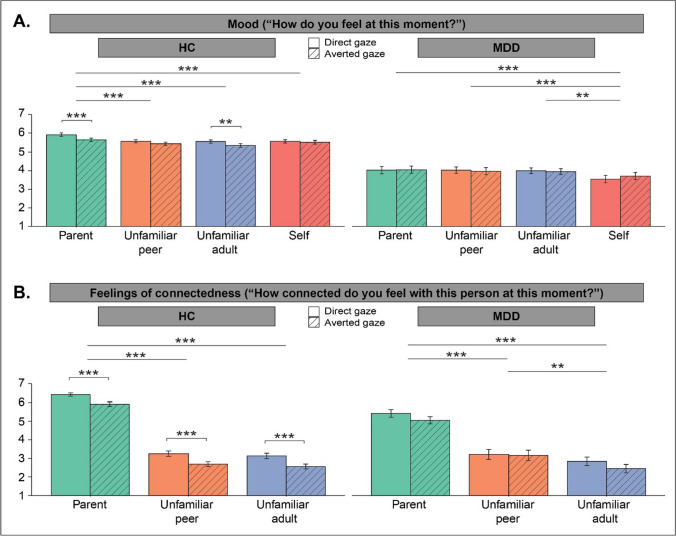
Mean levels of self-reported mood (**A**) and feelings of connectedness (**B**) after the videos of each target (i.e., parent, unfamiliar peer, unfamiliar adult, self) in either gaze direction (i.e., direct and averted) in HC (*n* = 59) and DEP adolescents (*n* = 19). Eye contact boosted mood and feelings of connectedness in HCs. Eye contact did not boost mood in depressed adolescents and did not report an improved mood and reported lower feelings of connectedness after seeing their parent relative to seeing unfamiliar others compared with HCs. Error bars represent standard error of the mean. Significance at *p < 0.05, **p < 0.01, and ***p < 0.001

Adolescents reported a better mood in response to direct versus averted gaze videos (*B* =  − 0.16, *SE* = 0.04, *t*(880) =  − 4.21, *p* < 0.001, *d* = 0.27, Fig. 2A left panel). In addition, adolescents’ mood was dependent on the target in the videos (χ^2^(3) = 42.77, *p* < 0.001). Bonferroni corrected post-hoc analyses revealed that they reported a better mood after videos of their parent versus an unfamiliar peer (*p* < 0.001, *d* = 0.47), unfamiliar adult (*p* < 0.001, *d* = 0.56), or themselves (*p* < 0.001, *d* = 0.40). Adolescents did not significantly differ in their mood after videos of an unfamiliar peer, unfamiliar adult, and themselves (all ≥ *p* = 0.597). There was no significant interaction between gaze direction and target on adolescents’ self-reported mood (*p* = 0.204).

##### Connectedness

Adolescents reported enhanced feelings of connectedness in response to direct versus averted gaze videos (*B* =  − 0.55, *SE* = 0.08, *t*(640) =  − 6.72, *p* < 0.001, *d* = 0.51; Fig. 2B, left panel). In addition, adolescents’ feelings of connectedness were dependent on the target in the videos (χ^2^(2) = 1406.24, *p* < 0.001), with adolescents feeling more connected with their parent versus an unfamiliar peer (*p* < 0.001, *d* = 2.95) or adult (*p* < 0.001, *d* = 3.06), but they did not differ in how connected they felt with an unfamiliar peer versus adult (*p* = 0.665, all Bonferroni corrected). There was no significant interaction between gaze direction and target on adolescents’ feelings of connectedness with others (*p* = 0.940).

To test whether prolonged eye contact boosted mood and feelings of connectedness relative to baseline, we examined adolescents’ self-reported affect in response to static pictures with direct gaze before the scan session with their averaged ratings after prolonged direct gaze videos of the targets during the task. These analyses indicated significant interactions between target and time point (i.e., pretask versus task) on adolescents mood (χ^2^(3) = 10.31, *p* = 0.016) and feelings of connectedness (χ^2^(2) = 70.50, *p* < 0.001). Adolescents did not report a mood-boosting effect in response to prolonged videos of others (versus static pictures) but reported a lower mood after being confronted with a prolonged video of their own direct gaze. In addition, adolescents reported enhanced feelings of connectedness in response to prolonged eye contact (versus static pictures), which was most pronounced in response to direct gaze videos of unfamiliar others compared to their parent (Supplement 5).

Responses to the question “How do you feel *about this person* at this moment?” were highly correlated with adolescents’ feelings of connectedness (*r* = 0.72, *p* < 0.001) and showed similar effects (Supplement 6).

### Gaze responses

**Fig. 3:**
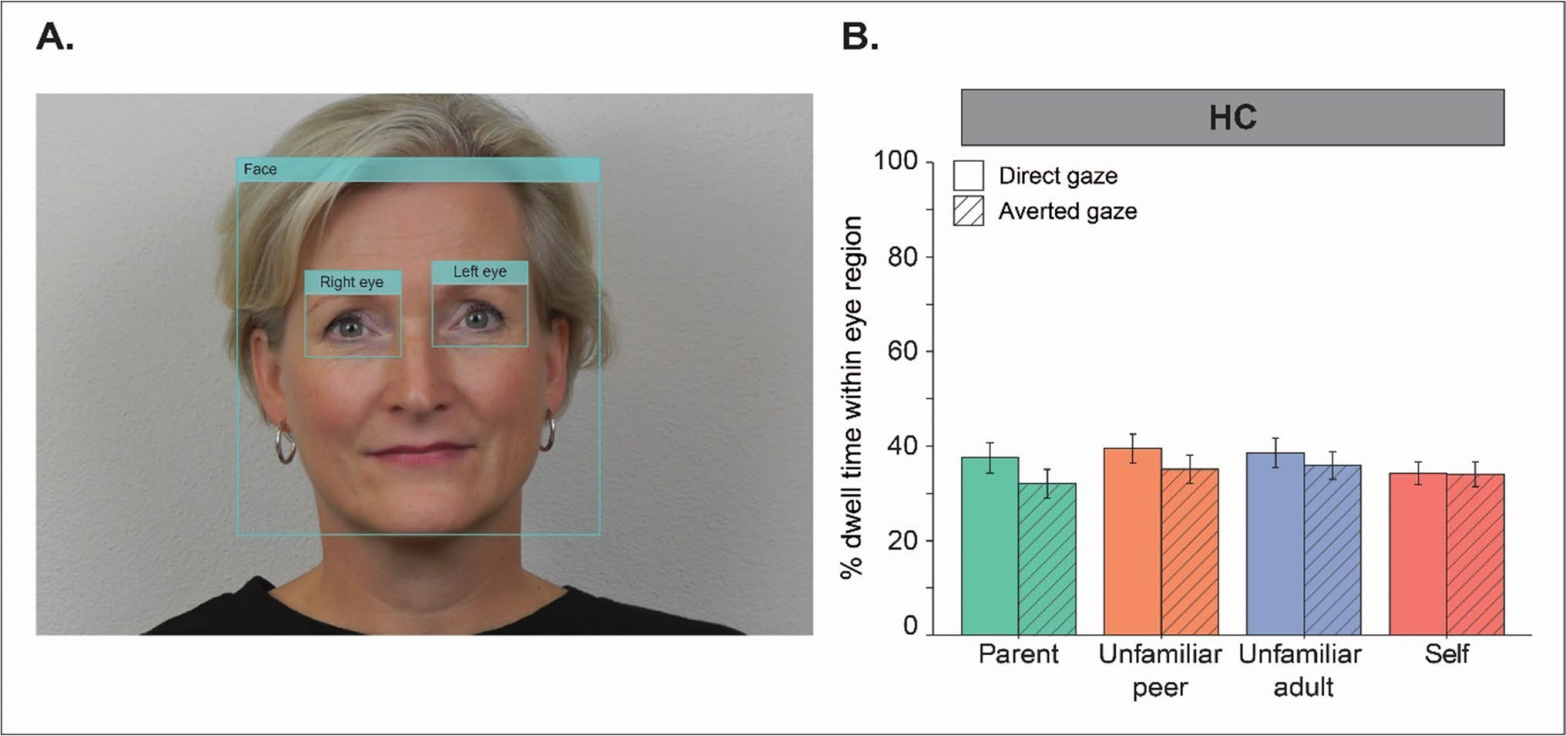
Average levels of gaze toward the eye region of targets (i.e., parent, unfamiliar peer, unfamiliar adult, self) in either gaze direction (i.e., direct and averted gaze) in HCs (*n* = 42). Gaze was operationalized as the percentage of dwell time toward the eye region relative to the total duration per video. The right and left eye AOIs were combined into a single AOI of the eye region (**A**). HC adolescents gazed more toward the eye region of targets during direct vs averted gaze videos (*B* = 3.09, *SE* = 1,22, t(611) 2.54, *p* = 0.011, *d* = 0.20). Gaze responses of HC adolescents were not affected by the identity of the targets in the videos

HC adolescents gazed more toward the eye region of targets during videos of direct versus averted gaze (*B* = 3.09, *SE* = 1.22, *t*(611) = 2.54, *p* = 0.011, *d* = 0.20; Fig. 3B, left panel). Our analyses (*n* = 42) did not reveal a main effect of target (*p* = 0.143), nor an interaction between gaze direction and target on adolescents’ gaze responses to the eye region of targets (*p* = 0.557).

### Neuroimaging findings

For all ROIs, we extracted BOLD responses to direct and averted gaze of the targets. We examined the effects of gaze direction, target, and their interaction, while controlling for multiple comparisons (*p* < 0.05/5) in five separate linear mixed regression model analyses.

**Fig. 4:**
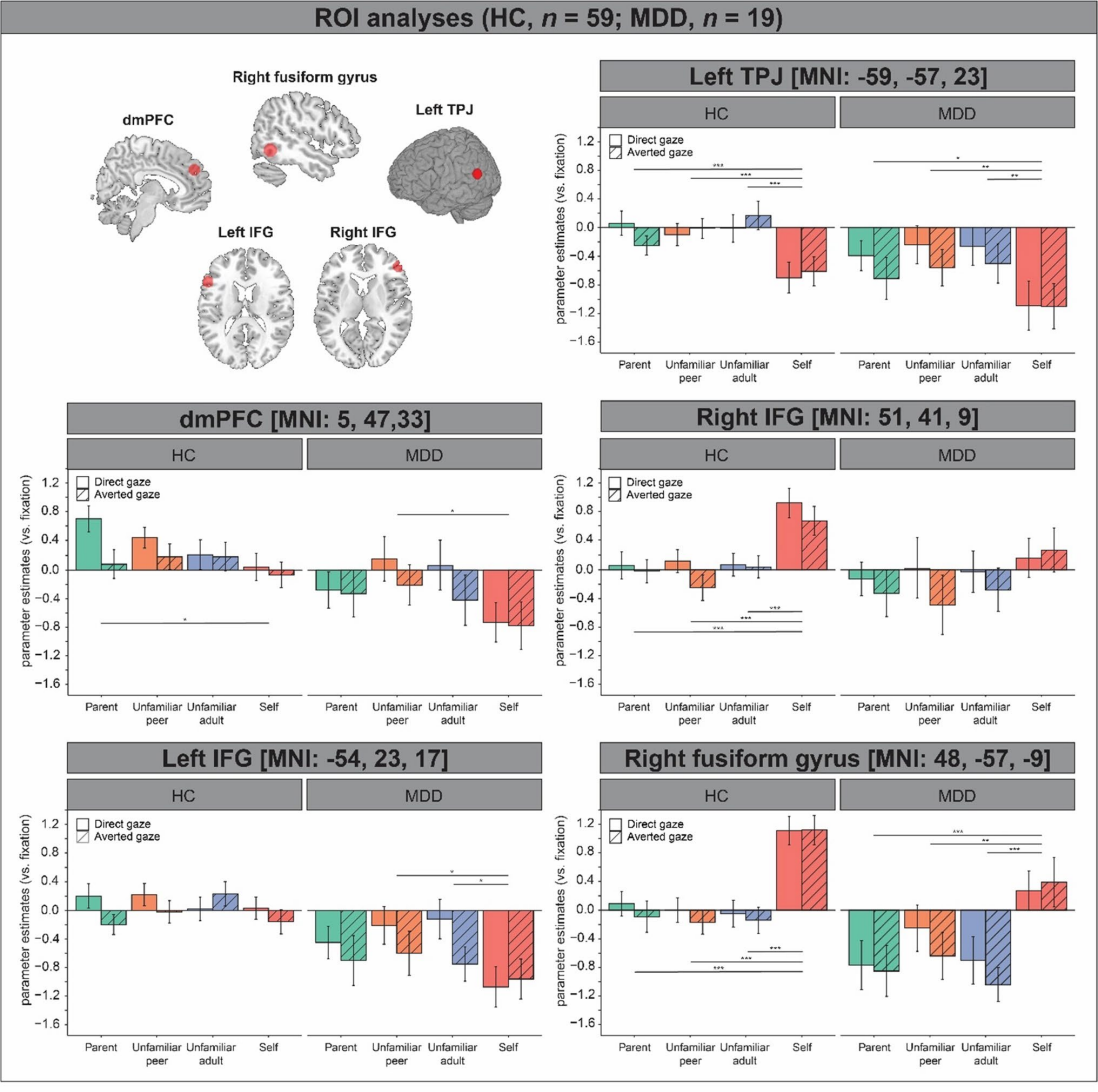
Overview of regions of interest (ROIs) in brain regions previously found to be involved in prolonged eye contact in adults and results showing how gaze direction and target modulated BOLD responses to prolonged eye contact with one’s parent, an unfamiliar peer or adult and the self in HC (*n* = 59) and DEP adolescents (*n* = 19). All ROIs were Bonferroni corrected for the number of tests (*p* < 0.05/5). Parameter estimates were contrasted against fixation in the eye contact task. Error bars represent standard error of the mean. Significance at *p < 0.05, **p < 0.01, ***p < 0.001

Our analyses in HC adolescents revealed a significant main effect of target in left TPJ (χ^2^(3) = 36.99, *p* < 0.001), right IFG (χ^2^(3) = 43.48, *p* < 0.001), and right FG (χ^2^(3) = 121.54, *p* < 0.001). Post-hoc (Bonferroni corrected) pairwise comparisons revealed decreased deactivation in BOLD response in left TPJ (parent: *p* < 0.001, *d* = 0.56; unfamiliar peer: *p* < 0.001, *d* = 0.60; unfamiliar adult: *p* < 0.001, *d* = 0.73) and decreased activation in BOLD response in right IFG (parent: *p* < 0.001, *d* = 0.68; unfamiliar peer: *p* < 0.001, *d* = 0.76; unfamiliar adult: *p* < 0.001, *d* = 0.65), and in right FG (parent: *p* < 0.001, *d* = 1.11; unfamiliar peer: *p* < 0.001, *d* = 1.20; unfamiliar adult: *p* < 0.001, *d* = 1.20) in response to “others” versus the self (Fig. 4, left panels). There were significant main effects of target (χ^2^(3) = 8.40, *p* = 0.038) and gaze direction (χ^2^(3) = 6.22, *p* = 0.013) in dmPFC, but these effects did not survive correction for multiple comparisons (*p* < 0.05/5). Analyses in other ROIs did not reveal additional main effects of gaze direction, nor there were significant interactions between gaze direction and target on adolescents’ neural responses in the eye contact task.

To test whether task-related BOLD activation in brain regions outside the ROIs was found in HC adolescents in response to direct and averted gaze of all targets, we performed a complementary whole-brain analysis. These analyses revealed a main effect of gaze direction in left superior frontal gyrus (SFG), right IFG, and left temporal pole and a main effect of target in right FG, right precentral gyrus, bilateral angular gyrus/TPJ, left middle occipital gyrus, right cuneus, right middle frontal gyrus, left middle temporal gyrus, left superior temporal gyrus. See Supplement 8–9 for details and post-hoc analyses on the directions of the whole-brain effects.

## Responses of DEP (versus HC) adolescents to prolonged eye contact (Goal 2)

### Affective responses

To examine differences in affective responses to eye contact between DEP and HC adolescents, we performed a generalized linear mixed regression analysis on the main effect of group (i.e., DEP versus HC) and its interactions with gaze direction and target on adolescents’ affect ratings.

#### Mood

Overall, DEP adolescents reported a significantly lower mood after the videos compared with HCs (main effect group: χ^2^(1) = 55.17, *p* < 0.001, *d* = 2.75; Fig. 2A). In addition, we found a significant interaction between group × target (χ^2^(3) = 19.82, *p* < 0.001). Whereas HCs reported a higher mood after videos of their parent versus an unfamiliar peer (*p* < 0.001, *d* = 0.46), an unfamiliar adult (*p* < 0.001, *d* = 0.53), or themselves (*p* < 0.001, *d* = 0.39), DEP adolescents reported a lower mood after videos of themselves versus their parent (*p* < 0.001, *d* = 0.68), an unfamiliar peer (*p* = 0.001, *d* = 0.61), and an unfamiliar adult (*p* = 0.003, *d* = 0.57). DEP adolescents did not show differences in self-reported mood in responses to videos of their parent versus an unfamiliar peer or adult. There also was a significant interaction between group × gaze direction (χ^2^(1) = 5.07, *p* = 0.024). Whereas HCs reported a better mood after videos with direct versus averted gaze (*p* < 0.001, *d* = 0.27), gaze direction did not affect the mood of DEP adolescents (*p* = 0.777). There was no significant interaction between group × gaze direction × target on adolescents’ mood (*p* = 0.829).

#### Connectedness

We found a significant interaction between group × target (χ^2^(2) = 31.04, *p* < 0.001), indicating that DEP (versus HC) adolescents reported to feel less connected with their parent (*p* = 0.021, *d* = 0.83), whereas they did not differ in feelings of connectedness in response to videos of an unfamiliar peer or adult (*p* = 1.000, for both). There was no main effect of group on adolescents’ feelings of connectedness (*p* = 0.234), nor a significant interaction between group × gaze direction (*p* = 0.103), indicating that DEP adolescents did not differ from HCs in how connected they felt in response to direct versus averted gaze videos. There also was no significant interaction between group × gaze direction × target on adolescents’ feelings of connectedness (*p* = 0.642).

### Neuroimaging findings

To examine differences in neural responses in DEP versus HC adolescents within the ROIs, we examined the main effect of group and their interactions with gaze direction and target on adolescents’ neural responses, while controlling for multiple comparisons (*p* < 0.05/5) in five separate mixed regression model analyses in *R*. These analyses revealed a main effect of group in left IFG (*B* = 0.65, *SE* = 0.24, *t*(76) = 2.68, *p* = 0.009, *d* = 0.73), showing diminished BOLD activation in this brain region in DEP versus HC adolescents, irrespective of gaze direction or target. There was also a main effect of group in dmPFC (*p* = 0.026) and right FG (*p* = 0.017), but these effects did not survive corrections for multiple comparisons. There were no significant interactions between group × target (all ≥ *p* = 0.207) or between group × gaze direction (all ≥ *p* = 0.204), nor a significant interaction between group × gaze direction × target (all ≥ *p* = 0.069) on adolescents’ neural responses in the ROIs.

To test differences in task-related BOLD activation in brain regions outside the ROIs between DEP and HC adolescents in response to direct and averted gaze of the targets, we conducted complementary whole-brain analyses. The analyses revealed an overall hypoactivation in left secondary visual cortex in DEP versus HC adolescents when performing the eye contact task (main effect of group: MNI peak-coordinate [− 8, − 87, − 9], *Z* = 5.23, *p*_cluster-level_ < 0.001, k = 4746). There were no significant interactions between group × gaze direction, group × target, or group × gaze direction × target.

### Neural responses to prolonged eye contact over time

As preregistered, we tested whether we could replicate a finding in adults who exhibited increased activation within the dmPFC with increasing duration of eye contact (MNI peak-coordinate: [2, 38, 47] (Wever et al., 2022). We performed a ROI analysis using an 8-mm spherical ROI around the peak-coordinate on the contrast of direct minus averted gaze videos pooled over all “other” conditions (i.e., parent, unfamiliar peer, unfamiliar adult) for HC adolescents. We split each trial into three epochs of equal length and subsequently tested for parametric increases and decreases with presentation duration [− 1 0 1]. We did not find a significant increase in BOLD activation over the course of prolonged eye contact duration in this region of the dmPFC in HC adolescents.

**Fig. 5:**
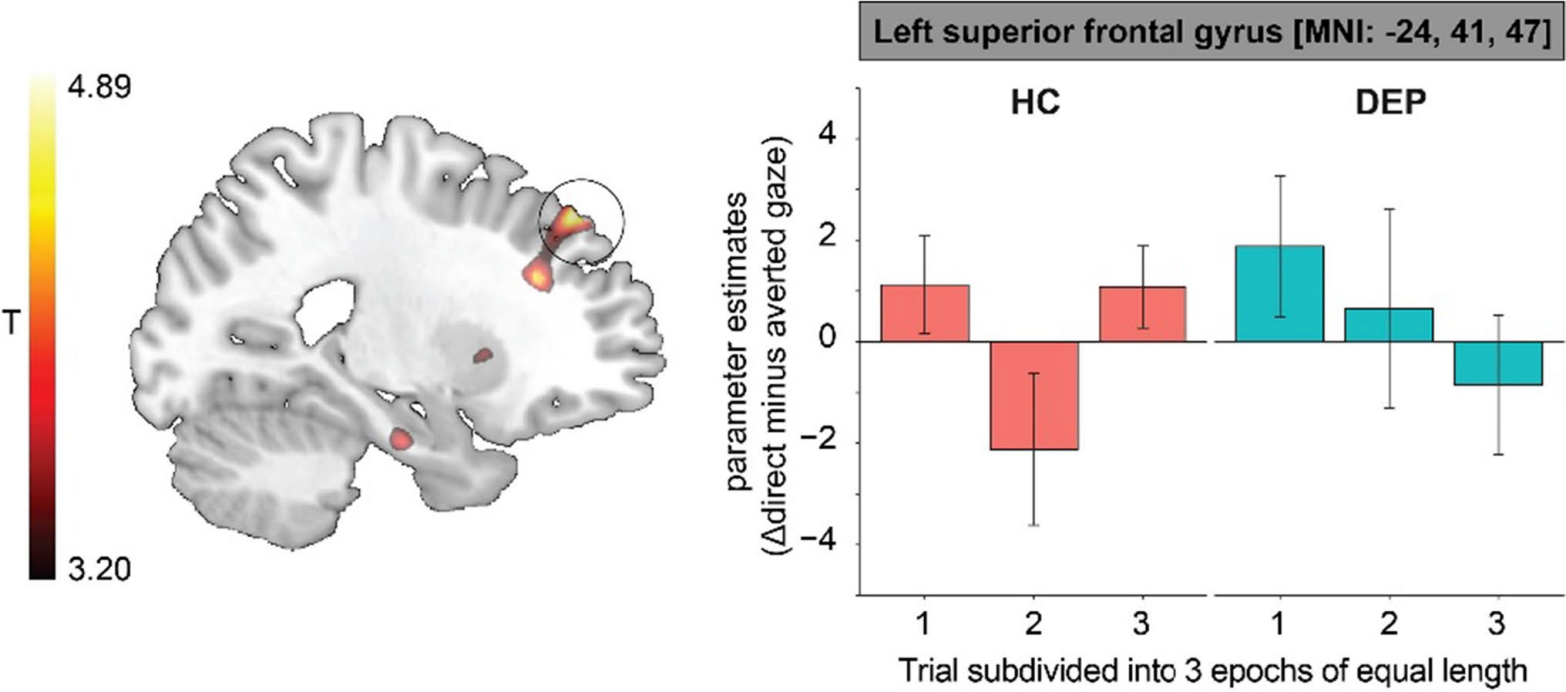
Prolonged eye contact was associated with parametric decreases over time in left superior frontal gyrus in DEP adolescents, whereas activation in this region was not associated with eye contact duration over time in HCs. We performed a parametric analysis testing for differences linear increases and decreases in neural responses associated with presentation duration of eye contact (Δdirect–averted gaze) between DEP and HC adolescents using a whole-brain approach. To visualize the parametric effect, we subdivide each video in 3 epochs of equal length and plotted average BOLD responses in left superior frontal gyrus for each epoch

Between-group differences in parametric increases and decreases of neural responses with increasing durations of eye contact were tested with an independent *t*-test between DEP and HC adolescents, using a whole-brain approach. We found a parametric decrease over time in left SFG in DEP adolescents, whereas activation in this region was not associated with eye contact duration over time in HCs (Fig. 5).

## Gaze data

The limited number of depressed adolescents from whom we successfully collected gaze data (*n* = 11 of 19) render these analyses likely to be underpowered since only relatively large effects can be detected with such a sample size. Results of these analyses can be found in Supplement 7 and need to be interpreted in the light of this modest sample size.

## Confound analyses

All outcomes remained significant after controlling for age, sex, and pubertal status of the adolescents. In addition, all outcomes at the neural level remained significant after controlling for handedness. These findings indicate that results were not driven by one of these variables (see Supplement 10 for details).

## Discussion

This study examined adolescents’ affective and neural responses to prolonged eye contact with their parent (Goal 1) and whether these responses differed in depressed adolescents (Goal 2). In response to eye contact, HC adolescents reported enhanced affective responses, made more eye contact, and showed enhanced neural responses in right IFG, left temporal pole, and left SFG, regardless of the target. Despite reporting a better mood and feeling more connected, and exhibiting enhanced neural responses to the sight of their parent versus unfamiliar others, gaze direction did not interact with adolescents’ responses to the distinct target identities at any response level. In contrast to HCs, eye contact did not induce a mood-boosting effect in depressed adolescents. While HCs reported increased mood and feelings of connectedness in response to the sight of their parent relative to the other targets, this effect was less strong in depressed adolescents. Furthermore, regardless of the target and gaze direction, depressed adolescents showed diminished neural activation in left IFG and left secondary visual cortex compared with HCs, suggesting overall blunted neural responses to the prolonged presentation of videos of self and others. Lastly, depressed adolescents showed decreased neural activation during the prolonged presentation of a direct over an averted gaze in left SFG, whereas this was not observed in HCs.

Consistent with findings in adults (Wever et al., 2022), prolonged eye contact (versus averted gaze) induced a better mood and feelings of connectedness in adolescents and substantial overlap in brain regions related to the processing of eye contact with others versus the self (i.e., left TPJ, right IFG, right FG, precentral gyrus, and left MOG). These are all brain regions that have been consistently found in socio-emotional processing and mentalizing (Herlin et al., 2021; Senju & Johnson, 2009), and have been linked to the communicative intent of eye contact (Cavallo et al., 2015). In addition, adolescents’ affective responses were strongest when they were presented with their own parent versus unfamiliar others, while they did not distinguish between the sight of an unfamiliar peer or adult. This aligns with research showing that parents are still *perceived* by the child as important advisors and continue to be important for their well-being (Baumrind, 1991; Steinberg & Silk, 2002; Yap et al., 2014). Remarkably, we did not find evidence for the engagement of a specific neural network in adolescents when presented with videos of their own parent versus others, indicating that this enhanced sensitivity to their parent was not reflected in their neural responses. Although the reason for this discrepancy is unclear, this is in contrast to parents who showed enhanced affective responses *and* decreased deactivation in left middle/inferior occipital gyrus and right IFG to the sight of their own child versus others (Wever et al., 2022).

The finding that depressed adolescents (versus HCs) reported a lower mood and diminished feelings of connectedness in response to the sight of their parent compared with nondepressed adolescents points toward a less positive perception of the parent in depressed adolescents. This is in line with numerous studies indicating that adolescent depression is associated with a lower perceived parent–child relationship quality (Branje et al., 2010; Restifo & Bögels, 2009; Sheeber et al., 2001; Yap et al., 2014) and with a lower self-reported parental care in depressed versus nondepressed adolescents in this study (Table 1). Several studies suggest that the directionality of this effect may go in both ways: Negative parenting behaviors, such as a lack of warmth and more critical parenting, are associated with increased depressive symptoms in adolescents over time, but adolescent’ depressive symptoms also are related to a lower perceived relationship quality with their parents (Branje et al., 2010; Heaven et al., 2004; Pavlidis & McCauley, 2001; Sheeber et al., 1997). As such, this emphasizes the importance of considering how adolescents *perceive* (eye) contact with their parents, especially when developing interventions to improve the parent–child relationship in families with a depressed adolescent.

Another remarkable finding in depressed versus nondepressed adolescents is the overall blunted BOLD activity in left IFG. This brain region has been consistently linked to human’s capacity to build and maintain attachment and the perception of shared internal states with others (Feldman, 2017) and might suggests that depressed adolescents might feel more alienated from others and themselves. This corroborates with findings that depression is characterized by negative thoughts about others (e.g., I cannot trust others) and the self (e.g., I am worthless), which in turn negatively affect their ability and/or willingness to engage in interpersonal contact (Beck, 2002; Garber et al., 1993). Moreover, it aligns with the finding that, in contrast to HCs, eye contact did not induce a mood-boosting effect and positive feelings about others in depressed adolescents and that depressed and nondepressed adolescents did not differ in their eye gaze patterns, indicating that they made equivalent eye contact with the targets. Hence, the blunted neural responses of depressed adolescents did not reflect differences in how well adolescents were able to follow the instruction to make eye contact with the targets.

Despite a better mood and more connectedness with the targets and more eye contact during videos with a direct versus an averted gaze, gaze direction did not interact with adolescents’ responses to the distinct target identities at any response level (i.e., affective, gaze, and neural responses). This was in contrast to our expectations and suggests that adolescents did not differentiate between making eye contact with their parents compared to unfamiliar others but only showed different responses to the sight of videos of their parents compared with others (irrespective of their gaze direction). Although we found enhanced activation in response to a direct gaze in right IFG, left temporal pole, and left SFG in exploratory whole-brain analyses, gaze direction did not modulate BOLD responses in our a priori defined ROIs. This is most likely due to the fact that ROIs were selected based on an analysis showing that neural activity in these ROIs was modulated by target identity, and not gaze direction, in the first and only published study using this paradigm (Wever et al., 2022). In this previously published study using this same paradigm, whole-brain analysis did not reveal brain regions that selectively responded to direct gaze versus averted gaze.

This study contributes to our understanding about adolescents’ perception of making eye contact with their parents and how this might differ in depressed adolescents. We used personally tailored stimuli of a prolonged duration of adolescents’ own parents and assessed a combination of subjective, gaze, and fMRI data. Nevertheless, this study is not without limitations. The task did not include a familiar peer or adult condition; therefore, we cannot rule out novelty effects introduced by the unfamiliar peer and adult conditions. However, presenting videos of a familiar peer might have introduced new complexities, such as variations in friendship quality. Our sample size of depressed adolescents was relatively small (*n* = 19) because of difficulties of recruiting depressed adolescents and their parents for an extensive fMRI study during the global COVID-19 pandemic. The null findings regarding the group differences between depressed and nondepressed adolescents may potentially reflect that effect sizes of group differences are too small to be detected with a sample size of 19 depressed adolescents. A post-hoc power analysis indicated that a sample size of 19 gives us 80% power to detect moderate to large effects with effect sizes of *r* ≥ 0.62. The majority of group differences between depressed and nondepressed adolescents in the literature is smaller, suggesting that our analyses may not be sufficiently powered to detect small and moderate effects. Inhomogeneity of variances between groups in affective and gaze responses also could have attributed to a lack of significant group differences. The assumption about homogeneity of variances between groups was not violated for neuroimaging analyses on a prior selected ROIs. Results regarding group differences should therefore be interpreted with caution and replicated in larger samples. In addition, it is of note that there was a skewed sex ratio in the group of depressed adolescents with 15 of 19 participants identifying as female. While this skewed sex ratio is representative of the high prevalence of depression amongst girls (Hankin & Abramson, 2001), it is unclear to what extent findings generalize to adolescent boys with depression. Finally, a potential concern about interpretation of our results relates to test–retest reliability of our fMRI task (Elliott et al., 2020; Noble et al., 2021). We performed a reliability analysis using a split-half approach where we calculated intraclass correlation coefficients (ICCs) for neural responses to each target condition (i.e., parent, unfamiliar peer, unfamiliar adult, self) in each run. ICCs ranged from 0.003–0.582, indicative of very modest test–retest reliability (see Supplement 11 for details). It should be noted that the order of magnitude of these reliability estimates is comparable to other studies using fMRI paradigms focused on emotional processing (Gee et al., 2015; Holiga et al., 2018; Kennedy et al., 2022; van den Bulk et al., 2013).

Our findings indicate that depressed adolescents do not benefit as much from the mood-boosting and connectedness-inducing effects of eye contact and have a less positive perception of the relationship with their parents compared to HCs. Moreover, they reported fewer positive feelings in response to others’ direct and averted gaze and exhibited diminished neural responses in a brain region related to attachment compared to HCs, suggesting a sense of alienation from others and self in depressed adolescents. Together these results contribute to our understanding of interpersonal difficulties in adolescents with depression, especially towards their parents and highlight the importance of including adolescents’ perception (among other perspectives) in interventions focusing on improvement of the parent–child bond in families with a depressed adolescent.

### Supplementary information

Below is the link to the electronic supplementary material.

Supplementary file1 (DOCX 1252 KB)

## Data Availability

The de-identified data, analysis scripts and materials for this study are available on DataverseNL and the MRI data are available on NeuroVault (https://neurovault.org/collections/FUOOKKIU/). All study measures, hypotheses, and analyses were preregistered at Open Science Framework prior to data analyses (https://osf.io/p6r28/). For any questions or additional material, please contact the corresponding author.
