# Supplementary material for: Neural and affective responses to prolonged eye contact with parents in depressed and nondepressed adolescents
Source: Cogn Affect Behav Neurosci. 2024 Feb 22;24(3):567–81. doi: 10.3758/s13415-024-01169-w (PMC11078816; doi:10.3758/s13415-024-01169-w)

**SUPPLEMENTARY MATERIALS**

**SUPPLEMENT 1**

***Details concerning the preparation of the video stimuli***

For the preparation of the video fragments, videos of adolescents and one of their parents were recorded during the lab visit. The videos had a minimal duration of 45s and were recorded in front of a white wall. Adolescents and parents wore a black t-shirt during the recordings to avoid distraction due to their clothing. We asked them to look in the camera with a friendly, but neutral, facial expression and to imagine looking into the eyes of their parent/child. They were also instructed not to stare, but to gaze as natural as possible and blinking was allowed. The individuals featured as the male and female unfamiliar peer and adult were selected based on age (between 11-17 years for the unfamiliar child and between 45-55 years for the unfamiliar adults) and sex. Videos were recorded under similar circumstances as videos of adolescents and parents who participated in the study and written informed consent was taken to confirm the approval of the targets to use their videos in the task.

All videos were presented twice in two separate runs (2 × 8 = 16 trials in total). During the first run, all targets were presented in a random order. For each target, adolescents were presented with two successive videos of the same target, but with gaze direction randomized (i.e., either starting with direct gaze or averted gaze, followed by the other direction). During the second run, the order of targets was randomized again, but the order of the presentation of the gaze direction was counterbalanced to the first run. The durations of the videos were based on a randomly chosen interval between 16-38s from prerecorded videos of 45s. The first and last 3s of each prerecorded video were discarded due to movement in the video recording. Stimuli of each condition were presented for a total duration of 54s across two repeats, meaning that duration of a stimulus in a specific condition in run 2 was 54s minus stimulus duration in run 1 with a minimum of 16s (range: 16-38s).

**SUPPLEMENT 2**

***fMRI data acquisition***

MRI images were acquired at the LUMC using a Philips 3.0T Achieva MRI scanner equipped with a SENSE-32 channel head coil. For the eye contact task, T2*-weighted echo planar imaging was used with the following parameters: TR = 2200 ms, TE = 30 ms, flip angle = 80°, FOV 220 × 220 × 114.7 mm, matrix size = 80 × 80, voxel size = 2.75 mm^3^, slice gap = 0.275 mm, 38 transverse slices in descending order. As subjective response ratings were self-paced, number of volumes varied between participants (run 1: M = 146.6, SD = 8.2, range = 126 – 165; run 2: M = 147.7, SD = 8.7, range = 129 – 172). A structural 3D T1 scan was acquired with the following parameters: TR = 7.9 ms, TE = 3.5 ms, TI = 820 ms, flip angle: 8°, voxel size = 1 mm^3^, 155 transverse slices FOV 195.8 × 250 × 170.5 mm, matrix size = 228 × 177, duration: 4:11 min. The first five volumes were discarded to allow for equilibration of T1 saturation effects. A b0 field map was acquired with the following parameters: TR = 200 ms, TE = 3.2, matrix size = 80 × 80, with 38 slices, voxel size = 2.75 mm^3^. The task was programmed and presented electronically using E-prime 2.0 (Tools Psychology Software, 2012) and participants could see the task through a mirror attached to the head coil. Foam inserts were used to restrict head motion if necessary.

**SUPPLEMENT 3**

**MNI coordinates of centers of mass of regions of interest**

| Brain regions of interest (ROIs) | | MNI coordinates | | |
| --- | --- | --- | --- | --- |
|  | X | | y | z |
| Left temporoparietal junction (TPJ) | -59 | | -57 | 23 |
| Dorsomedial prefrontal cortex (dmPFC) | 5 | | 47 | 33 |
| Left inferior frontal gyrus (IFG) | -54 | | 23 | 17 |
| Right inferior frontal gyrus (IFG) | 51 | | 41 | 9 |
| R fusiform gyrus (FG) | 48 | | -57 | -9 |

*Note.* The MNI coordinates used in this study were selected based on previously found regions using the same eye contact task in adults (Wever et al., 2022).

**SUPPLEMENT 4**

**Questionnaire details**

*Patient Health Questionnaire (PHQ-9)*

Adolescents’ severity of depressive symptoms was assessed with the Patient Health Questionnaire (PHQ-9) after the scan session (62). The questionnaire includes nine items corresponding with the nine symptoms of a major depressive disorder of the DSM-IV. Adolescents were asked to assess how much they were bothered by the symptoms over the past two weeks. Answers were given on a Likert scale ranging from 0 (*not at all*) to 3 (*nearly every day*). The sum score could range from 0-27, with higher scores representing a higher depression severity. The PHQ-9 has been validated and internal consistency and test-retest reliability are excellent and criterion, construct and external validity were established (62). PHQ-9 scores of ≥ 15 were indicated as within the clinical range. The internal consistency in the current sample was α = 0.92. Sum scores were log-transformed to account for skewness.

*Screen for Child Anxiety Related to Emotional Disorders (SCARED)*

Adolescents’ severity of anxiety symptoms was assessed with an adapted version of the Screen for Child Anxiety Related Emotional Disorders for (SCARED) prior to the first assessment day (Birmaher et al., 1997; Muris et al., 2007). This version was based on the original 71-item version of the SCARED (Bodden et al., 2009), and includes 31 items from the subscales panic disorder, generalized anxiety disorder, and social anxiety. Adolescents were asked to assess to what extent the sentences were reflecting their current situation. Answers were given on a Likert-scale from 0 (*Not true or hardly even true*) to 2 (*very true or often true*). The total score is computed by summing up all items and could range from 0-62, with higher scores representing a higher anxiety severity. A total score of ≥ 20 was indicated as within the clinical range. The internal validity and reliability have been established (Birmaher et al., 1997). The internal consistency in the current sample was α = 0.94.

*Parental Bonding Instrument (PBI)*

The adolescents’ perspective on the parent-child bond was assessed with the Parental Bonding Instrument (PBI) prior to the first assessment day (Parker et al., 1979). Adolescents were asked to report on perceived parenting styles of the parent of whom videos were shown in the eye contact task. The instrument consists of three subscales: parental care (12 items), parental overprotection (6 items), and autonomy support (6 items) of parents. One item of the autonomy support scale (“my mother/father don’t want me to grow up”) was left out due to a programming issue in the questionnaire. A total score and a score per subscale were computed for all adolescents by summing all items. The internal validity and reliability have been established (Arrindell et al., 1998; Enns et al., 2002). The internal consistency of the PBI in the current sample was α = 0.68 for the total score, α = 0.87 for the parental care, α = 0.66 for overprotection, and α = 0.78 for autonomy support.

*Edinburgh Handedness Inventory (EHI)*

Handedness was assessed by a modified 10-item version of the Edinburgh Handedness Inventory (EHI) developed by Oldfield (1971). The self-report questionnaire consists of ten questions about which hand is used during specific actions and answer categories were always left (−100), most times left (-50), both (0), most times right (50), and always right (100). Sum scores were calculated by the following formula for the laterality quotient (LQ): [R-L]/[R + L] ∗ 100 and ranged from +100 (right-handedness in all tasks) to −100 (left-handedness in all tasks). To convert the continuous laterality quotient into a dichotomous variable of right- and left-handedness that was used to control for in analyses we used the cut-off score of zero with quotients > 0 indicating right-handedness and < 0 indicating left-handedness. In the current sample, 72 adolescents (n = 18 DEP, n = 54 HC) were right-handed (92.3%) and 6 adolescents (n = 1 DEP, n = 5 HC) were left handed (7.7%).

*Pubertal Development Scale (PDS)*

Pubertal status was assessed by the Pubertal Development Scale (PDS), prior to the first assessment day (Petersen et al., 1988). This self-report measure consists of 5 questions about secondary sexual characteristics, including growth spurt, body hair, changes in the skin, for boys a question about changes in voice and facial hair, and for girls a question about breast development and menarche. Answers were given on a four-point scale: (1) had not yet started to develop, (2) was showing the first signs of development, (3) was showing clear development, or (4) had already finished developing. Scores were averaged to create a composite pubertal status score.

**SUPPLEMENT 5**

**
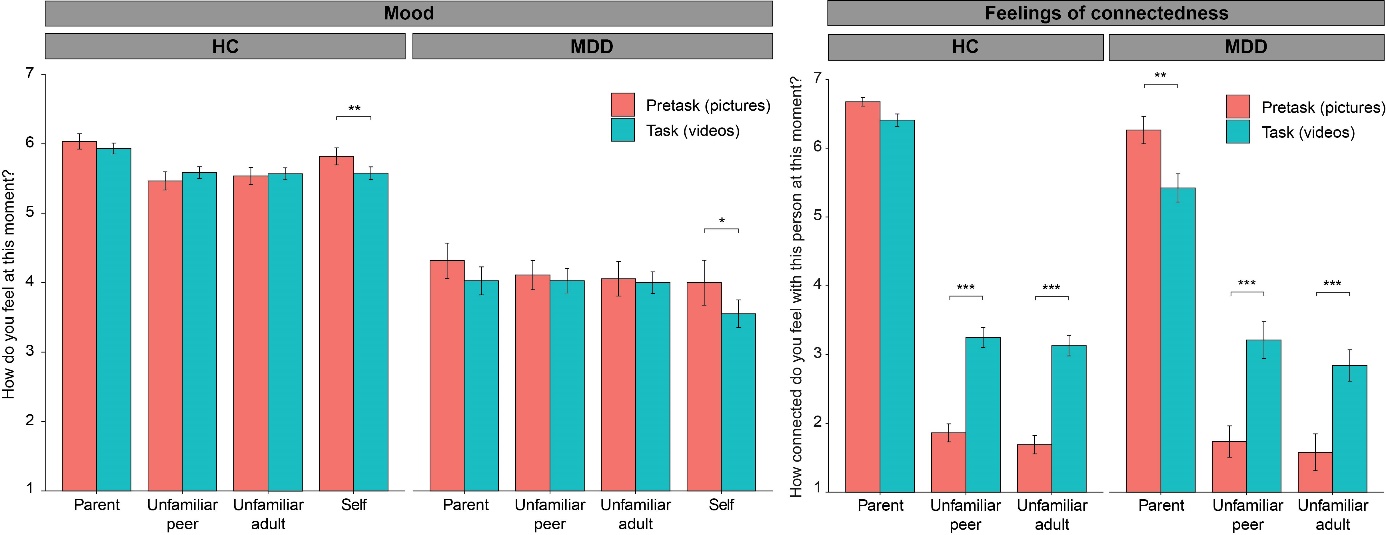
**

**Self-reported affective responses of adolescents with respect to mood and feelings of connectedness after seeing a static picture of each target prior to the task (pre-task) and in response to direct gaze video stimuli in the scanner.** Dynamic videos of prolonged eye contact, in contrast to static pictures, enhanced adolescents’ feelings about the targets and their feelings of connectedness, but it did not enhance adolescents’ mood. Error bars represent standard error of the mean. Significant *p*-values <.05 were indicated by *, *p* <.01 by **, and *p* <.001 by ***.

**SUPPLEMENT 6**

**Adolescents’ feelings about the targets.**

After watching each video, adolescents were asked to answer three questions; (1) “How connected do you feel with this person at this moment?”, (2) “How do you feel about this person at this moment?”, and (3) “How do you feel at this moment?”. Responses to the question “How do you feel about this person at this moment?” were highly correlated with adolescents’ feelings of connectedness (*r*=.72, *p*<.001) and showed similar effects, which are presented here in this supplement. We performed a generalized linear mixed regression model with gaze direction and target on adolescents’ affect ratings to the question “how do you feel about this person at this moment?”.

HCs reported more positive feelings about the targets (*B*=-0.42, *SE*=0.06, *t*(642)=-7.44, *p*<.001, *d*=0.56) in response to direct versus averted gaze videos. In addition, adolescents’ feelings about the targets were dependent on the target in the videos (*χ^2^*(2)=750.18, *p*<.001). Bonferroni corrected post hoc analyses revealed that they felt more positive about their parent versus an unfamiliar peer (*p*<.001, *d*=2.17) or adult (*p*<.001, *d*=2.20), but did not significantly differ in how they felt about an unfamiliar peer vs adult (*p*=1.000). There was no significant interaction between gaze direction × target on adolescents’ feelings about the targets with the others (*p*=.689).

Regarding differences in adolescents’ feelings about the targets between DEP and HC adolescents, we found a significant interaction between group × target (χ^2^(2)=29.98, *p*<.001). DEP adolescents reported to feel less positive about their parent compared to HC adolescents (*p*=.002), while no differences were found in how positive they felt about an unfamiliar peer or adult in response to their videos (*p*=1.000, for both). In addition, we found a significant interaction between group × gaze direction (χ^2^(1)=4.62, *p*=.032), showing that DEP adolescents did not significantly differ in how positive they felt about the targets after direct vs averted gaze videos (*p*=.172), while HC adolescents did feel more positive about the targets after direct vs averted gaze videos (*p*<.001). Lastly, DEP adolescents reported to feel less positive about the targets in general compared to HC adolescents (χ^2^(1)=4.18, *p*=.041). There was no significant three-way interaction between group × gaze direction × target on adolescents’ feelings about the targets (*p*=.437).


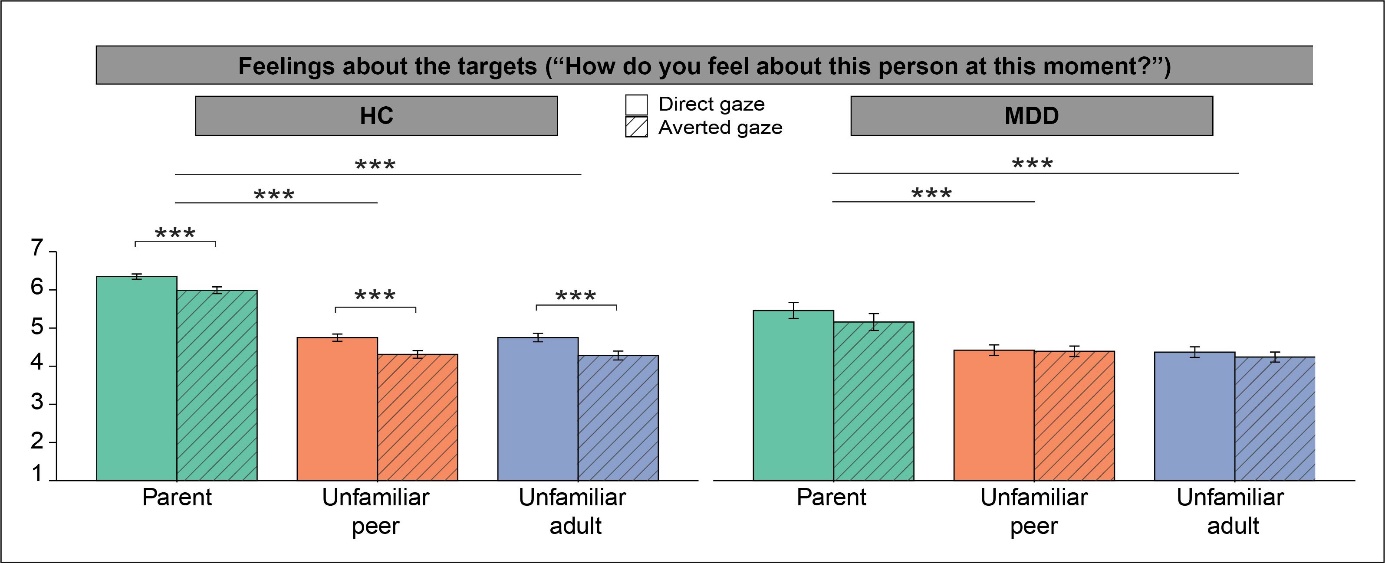


**SUPPLEMENT 7**

***Gaze responses towards the eye region of targets in either gaze direction in depressed and non-depressed adolescents.***

To examine differences in gaze responses between DEP (*n* = 11) and HC (*n* = 42) adolescents, we performed a generalized linear mixed regression model resting for a main effect of group, and interactions between group and gaze direction and target on adolescents’ percentage of gaze to the eye region of targets relative to the total video durations. There was no significant main effect of group (*p* = .278), indicating that DEP and HC adolescents did not differ in eye gaze behavior towards the eye region, irrespective of gaze direction or target. Also, there was no significant interaction between group × target (*p* = .491), group × gaze direction ( *p* =.154), or between group × target × gaze direction (*p* = .441). Gaze results should be interpreted in the light of the small number of depressed adolescents (n = 11) as only relatively large effects would be detectable with this sample size.


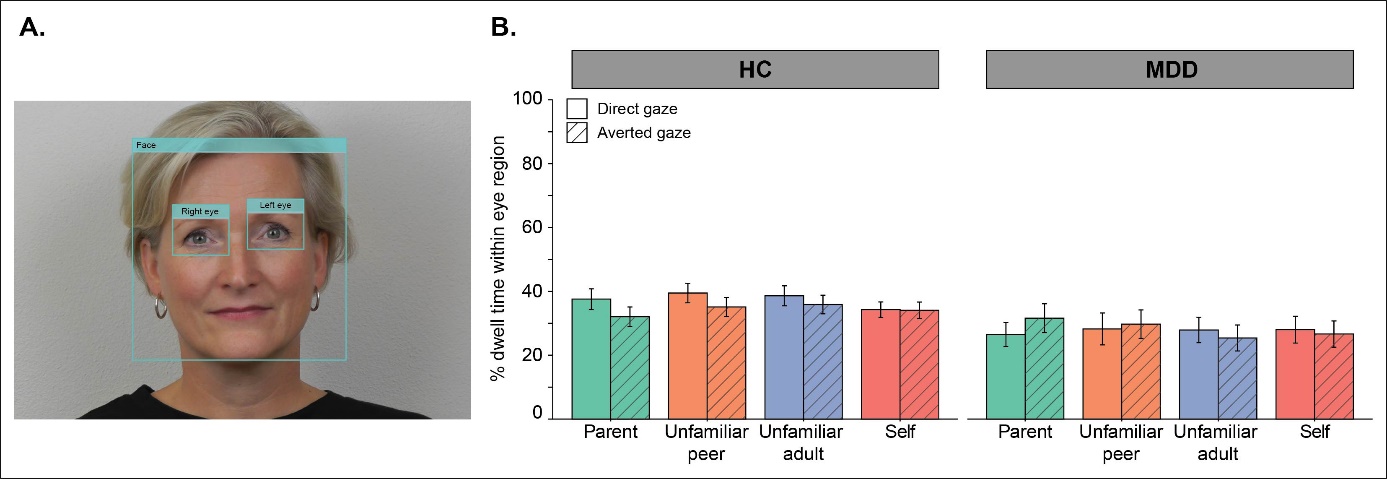


**SUPPLEMENT 8**

***Neuroimaging findings: Whole-brain results in HC adolescents***

To test whether task-related BOLD-activation in brain regions outside the ROIs was found in HC adolescents in response to direct and averted gaze of all targets, we performed a complementary whole-brain analysis. This analysis revealed a significant main effect of gaze direction in left superior frontal gyrus (extending into dmPFC), right inferior frontal gyrus (into dlPFC), and left temporal pole, indicating enhanced BOLD-responses to direct versus averted gaze videos.

In addition, there was a main effect of target in right fusiform gyrus (overlap with right fusiform gyrus ROI), right precentral gyrus, bilateral TPJ (overlap with left TPJ ROI), left middle occipital gyrus, right cuneus, right middle frontal gyrus/dlPFC (overlap with right IFG ROI), left middle temporal gyrus, left superior temporal gyrus. Post-hoc pairwise (Bonferroni corrected) comparisons indicated decreased deactivation to videos of their parent vs an unfamiliar adult in right cuneus (*p*=.003), right TPJ (*p*<.001), left TPJ (*p*=.006), right superior temporal gyrus (*p*=.033), and left middle temporal gyrus (*p*=.018). In addition, we found enhanced activation to videos of an unfamiliar adult versus their parent in left middle occipital gyrus (p=.003), right fusiform gyrus (*p*<.001), right precentral gyrus (*p*=.030), and right middle frontal gyrus/dlPFC (*p*=.023). Regarding adolescents’ neural responses to videos of an unfamiliar peer versus an unfamiliar adult, we found decreased deactivation in right cuneus (*p*<.001), right TPJ (*p*<.001), and left superior temporal gyrus (*p*=.011) and enhanced activation to an unfamiliar adult vs unfamiliar peer in left middle occipital gyrus (*p*<.001), right fusiform gyrus (*p*<.001), right precentral gyrus (*p*<.001), and right middle frontal gyrus/dlPFC (*p*=.014). Adolescents did not show any significant differences in BOLD-responses between parents and unfamiliar peers.

Regarding the self, adolescents showed diminished activation to videos of the self vs their parent in right cuneus (*p*=.026), right TPJ (*p*=.001), and right middle temporal gyrus (*p*=.020) and enhanced activation in right fusiform gyrus (*p*=.008). In addition, adolescents showed diminished activation to videos of the self vs an unfamiliar peer in right cuneus (*p*=.006) and right TPJ (*p*=.001) and showed enhanced activation in left middle occipital gyrus (*p*<.001) and right fusiform gyrus (*p*<.001). Adolescents did not significantly show altered BOLD-responses to videos of an unfamiliar adult vs the self.

There was no significant interaction between gaze direction × target on adolescents’ neural responses to eye contact at whole brain level.

**
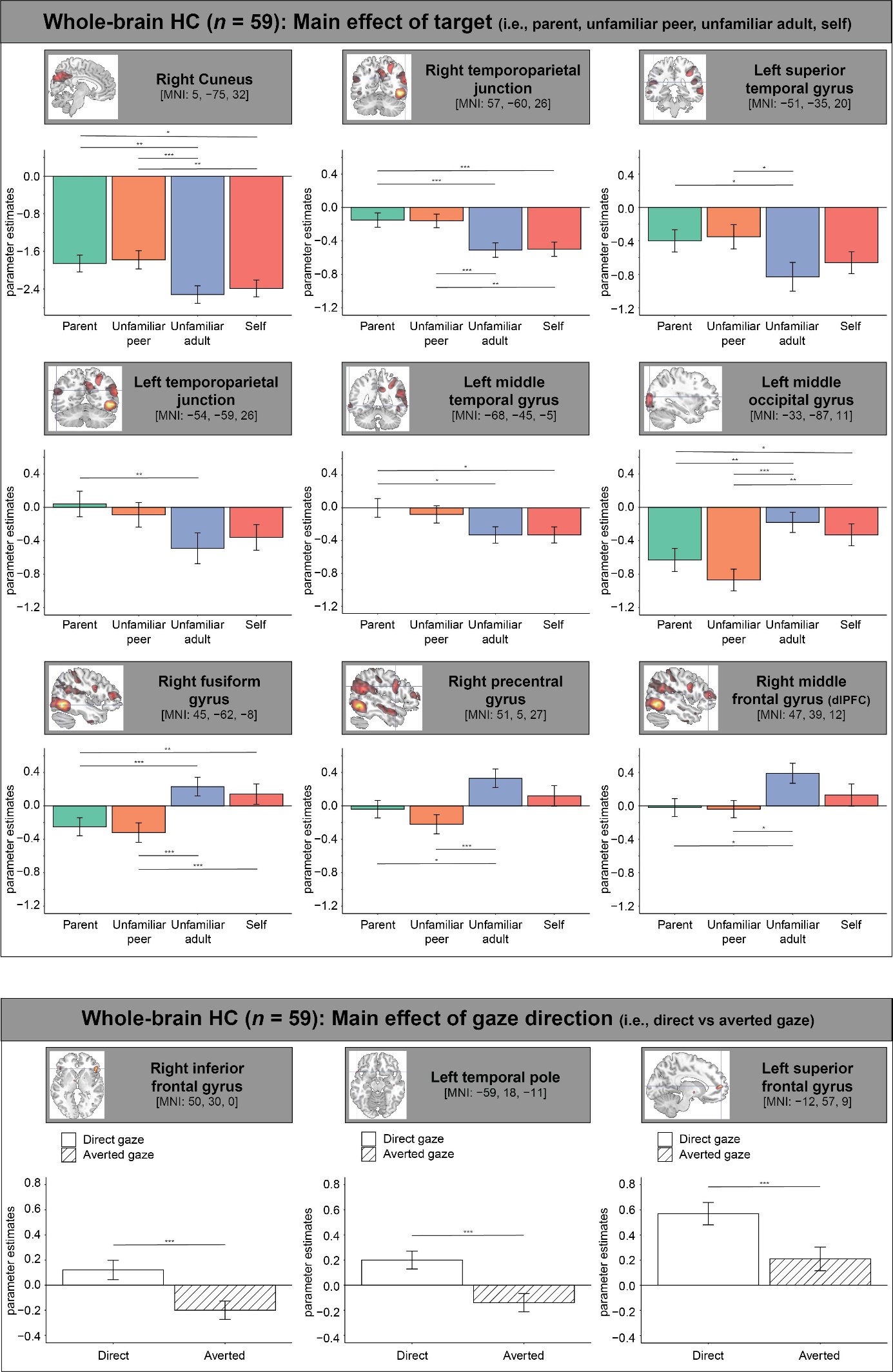
**

**SUPPLEMENT 9**

Peak activation coordinates of healthy control adolescents (*n* = 59) at whole-brain level for main effects of target and gaze direction.

|  |  | | | Voxel  test value | Cluster *p*-value | Cluster size |
| --- | --- | --- | --- | --- | --- | --- |
| Brain regions | **MNI-coordinates** | | |  |  |  |
|  | **x** | **y** | **z** | ***Z*** |  |  |
| Target | | | | | | |
| R Fusiform gyrus | 45 | -62 | -8 | ≥8 | <.001 | 12900 |
| R Middle occipital gyrus | 36 | -81 | 6 | 7.55 |  |  |
| R Superior parietal gyrus | 24 | -63 | 50 | 5.93 |  |  |
| R Precentral gyrus | 51 | 5 | 27 | 6.09 | .003 | 1053 |
| R Angular gyrus (TPJ) | 57 | -60 | 26 | 6.00 | <.001 | 10283 |
| R Middle temporal gyrus | 69 | -33 | 0 | 5.87 |  |  |
| R Superior temporal gyrus | 53 | -50 | 23 | 5.86 |  |  |
| L Middle occipital gyrus | -33 | -87 | 11 | 5.89 | <.001 | 3338 |
| L Fusiform gyrus | -47 | -69 | -9 | 5.18 |  |  |
| R Cuneus | 5 | -75 | 32 | 5.79 | <.001 | 5873 |
| R Posterior cingulate cortex, ventral part | 14 | -53 | 32 | 5.41 |  |  |
| L Cuneus | -8 | -78 | 27 | 4.25 |  |  |
| R Middle frontal gyrus (dlPFC) | 47 | 39 | 12 | 5.47 | .001 | 1185 |
| R Middle frontal gyrus | 44 | 59 | 8 | 3.22 |  |  |
| L Middle temporal gyrus | -68 | -45 | -5 | 4.56 | .013 | 768 |
| L Middle temporal gyrus | -65 | -38 | -6 | 4.09 |  |  |
| L Middle temporal gyrus | -57 | -32 | -6 | 3.56 |  |  |
| L Superior temporal gyrus | -51 | -35 | 20 | 4.23 | .004 | 1005 |
| L Superior temporal gyrus | -63 | -26 | 11 | 3.58 |  |  |
| L Angular gyrus (TPJ) | -54 | -59 | 26 | 4.17 | .005 | 962 |
| Gaze direction | | | | | | |
| L Superior frontal gyrus, medial part | -12 | 57 | 9 | 4.46 | .006 | 1100 |
| L Superior frontal gyrus, medial part | -3 | 57 | 17 | 3.98 |  |  |
| L Superior frontal gyrus (dmPFC) | -6 | 54 | 26 | 3.62 |  |  |
| R Inferior frontal gyrus, triangular part | 50 | 30 | 0 | 4.27 | .005 | 1135 |
| R Inferior frontal gyrus, opercular part | 48 | 18 | 8 | 3.99 |  |  |
| R Inferior frontal gyrus (dlPFC) | 51 | 24 | 18 | 3.56 |  |  |
| L Temporal pole | -59 | 18 | -11 | 4.03 | <.001 | 1873 |
| L Temporal pole | -41 | 23 | -24 | 3.97 |  |  |
| L Temporal pole | -50 | 14 | -39 | 3.84 |  |  |

*Note.* The Automated Anatomical Labeling atlas (AAL3) by Rolls et al. (2019) was used to label the peak coordinates.

**SUPPLEMENT 10**

To check whether results were not driven by differences in age, sex, and pubertal status of adolescents, we performed additional analyses to control for these variables by individually adding the covariates to the existing models. The neural ROI and whole-brain analyses were additionally controlled for left-handedness.

*Affective responses*

Adding age, sex, and pubertal status to the models analyzing adolescents’ affective responses did not result in different outcomes, indicating that these results were independent of these variables. Assessing the main effects of these covariates showed that age was not significantly associated with feelings about the targets in the videos (*p* = .371) or mood (*p* = .072), but adolescents who were older reported to feel less connected with the targets in the videos compared to younger adolescents (*B* = -0.18, SE = .08, *t*(75) = -2.14, *p* = .036). Sex was not significantly associated with self-reported level of connectedness (*p* = .644), feelings about the targets in the videos (*p* = .096), or own mood (*p* = .186). Pubertal status was also not significantly associated with self-reported levels of connectedness (*p* = .214), feelings about the targets in the videos (*p* = .545), or own mood (*p* = .072).

*Gaze responses*

Adding age, sex, and pubertal status to the models analyzing adolescents’ gaze responses did not result in different outcomes, indicating that these results were independent of these variables. Assessing the main effects of these covariates showed that both age and sex of adolescents was not significantly associated with the total duration of gaze towards the eye region of the targets (*p* = .061 and *p* = .246, respectively). However, a more advanced pubertal status (assessed with the pubertal development scale, PDS) was associated with more eye contact with the targets in the videos (*B* = 9.60, SE = 4.16, *t*(50) = 2.31, *p* = .025).

*Neural responses*

Regarding analyses of brain activity, all results remained significant and only minor changes in peak coordinates (whole-brain analyses) were observed when taking adolescents’ age, sex, pubertal status, or left-handedness into account. None of the covariates was significantly associated with neural activation assessed in the whole-brain model. Regarding the individual ROI analyses, there were no main effects of sex on BOLD-responses in any of the ROIs (left TPJ: *p* = .598, dmPFC: *p* = .999, left IFG: *p* = .365, right IFG: *p* = .721, right FG: *p* = .244) and no main effects of pubertal status on BOLD-responses in any of the ROIs (left TPJ: *p* = .084, dmPFC: *p* = .520, left IFG: *p* = .697, right IFG: *p* = .577, right FG: *p* = .680). There were also no main effects of left-handedness on BOLD-responses in any of the ROIs (left TPJ: *p* = .256, dmPFC: *p* = .837, left IFG: *p* = .166, right IFG: *p* = .617, right FG: *p* = .293). There was a main effect of age on adolescents’ BOLD-responses in right IFG, showing that older adolescents exhibited lower BOLD-responses in right IFG compared to younger adolescents (*B* = -0.15, SE = 0.08, *t*(75) = -2.01, *p* = .048). There were no main effects of age on BOLD-responses in the other ROIs (left TPJ: *p* = .085, dmPFC: *p* = .061, left IFG: *p* = .114, right FG: *p* = .057).

**SUPPLEMENT 11**

To examine split-half reliability between runs we calculated intraclass correlation coefficients (ICCs) per participant per run, and also per target condition (i.e., parent, unfamiliar peer, unfamiliar adult, self). Individual contrast values of run 1 and run 2 were used for all five ROIs and the strongest five whole-brain results for which we built 8-mm ROI spheres around the peak-coordinates. ICCs were calculated in R with the “irr” package. We followed the guidelines proposed by Cicchetti for qualifying reliability: poor (<0.40), fair (0.41-0.59), good (0.60-0.74), or excellent (>0.75) (Cicchetti, 2001; Cicchetti & Sparrow, 1981).

As can be seen in the figure below, ICCs per brain region ranged from 0.003-0.582 and were highly dependent on the target person in the stimuli.


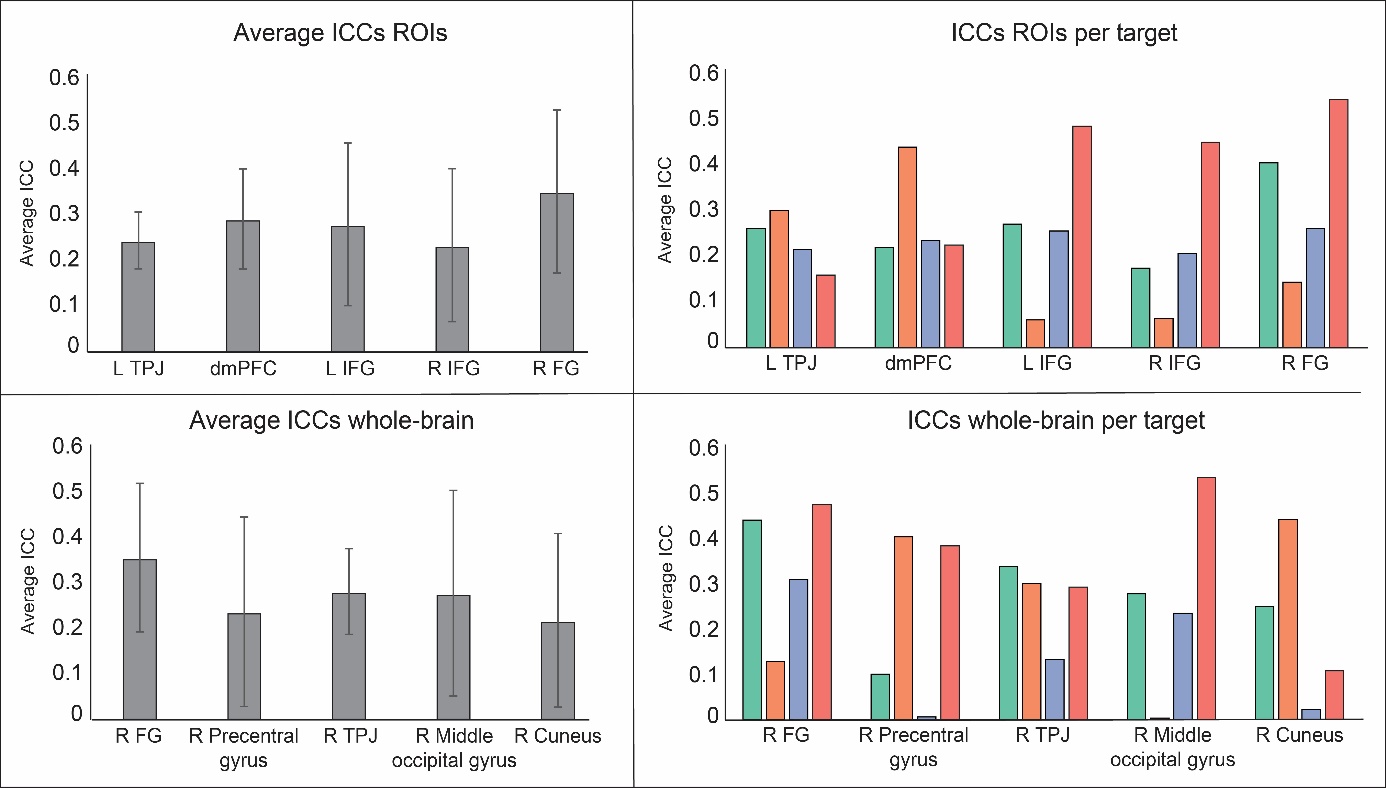

Supplement: Supplementary file 1 — Supplementary file1 (DOCX 1252 KB) [file 13415_2024_1169_MOESM1_ESM.docx]
